# Marfan Syndrome Versus Bicuspid Aortic Valve Disease: Comparative Analysis of Obstetric Outcome and Pregnancy-Associated Immediate and Long-Term Aortic Complications

**DOI:** 10.3390/jcm9041124

**Published:** 2020-04-15

**Authors:** Betül Toprak, Katalin Szöcs, Elvin Zengin-Sahm, Christoph Sinning, Amra Hot, Peter Bannas, Kurt Hecher, Bernd Hüneke, Thomas S. Mir, Meike Rybczynski, Evaldas Girdauskas, Stefan Blankenberg, Yskert von Kodolitsch

**Affiliations:** 1 Department of Cardiology, University Heart and Vascular Center Hamburg, University Medical Center Hamburg-Hospital Eppendorf, 20246 Hamburg, Germany; betuel.toprak@uke.de (B.T.); k.szoecs@uke.de (K.S.); e.zengin@uke.de (E.Z.-S.); c.sinning@uke.de (C.S.); m.berner@uke.de (M.R.); s.blankenberg@uke.de (S.B.); 2 Department of Medical Biometry and Epidemiology, University Medical Center Hamburg-Hospital Eppendorf, 20246 Hamburg, Germany; a.hot@uke.de; 3 Department of Diagnostic and Interventional Radiology, University Medical Center Hamburg-Hospital Eppendorf, 20246 Hamburg, Germany; p.bannas@uke.de; 4 Department of Obstetrics and Fetal Medicine, University Medical Center Hamburg-Hospital Eppendorf, 20246 Hamburg, Germany; k.hecher@uke.de (K.H.); hueneke@uke.de (B.H.); 5 Department of Pediatric Cardiology, University Heart and Vascular Center Hamburg, University Medical Center Hamburg-Hospital Eppendorf, 20246 Hamburg, Germany; mir@uke.de; 6 Department of Cardiovascular Surgery, University Heart and Vascular Center Hamburg, University Medical Center Hamburg-Hospital Eppendorf, 20246 Hamburg, Germany; e.girdauskas@uke.de

**Keywords:** Marfan syndrome, bicuspid aortic valve, aortopathy, aortic dissection, pregnancy

## Abstract

Pregnancy poses a threat to women with aortopathy. Conclusive data on the obstetric and aortic outcome in this risk collective, especially when it comes to aortic complications in the long term, are still missing. This study offers a comparative analysis of pregnancy-associated outcome in 113 consecutive women with Marfan syndrome or bicuspid aortic valve disease, including 46 ever-pregnant and 37 never-pregnant women with Marfan syndrome, and 23 ever-pregnant and 7 never-pregnant females with bicuspid aortic valve disease. The overall obstetric outcome was comparable between ever-pregnant women with Marfan syndrome and with bicuspid aortic valve disease (*p* = 0.112). Pregnancy-associated aortic dissection occurred in two women with Marfan syndrome (3%) during a total of 62 completed pregnancies, whereas no single case of aortic event occurred in women with bicuspid aortic valve disease during a total of 36 completed pregnancies (*p* = 0.530). In the long-term follow-up, aortic dissection occurred in 21% of ever-pregnant women with Marfan syndrome, but in none of the women with bicuspid aortic valve disease (*p =* 0.022). Proximal aortic surgery was performed with similar frequency in ever-pregnant women with Marfan syndrome and with bicuspid aortic valve disease in the long term (*p* = 0.252). However, ever-pregnant women with Marfan syndrome were younger when surgery was performed (44 ± 9 vs. 59 ± 7 years; *p* = 0.041). In Marfan syndrome, long-term growth of the aorta was comparable between ever-pregnant and never-pregnant women. Pregnancy thus exhibited an increased immediate aortic risk only in women with Marfan syndrome, but not in women with bicuspid aortic valve disease. Previous pregnancy did not relate to an increased long-term risk of adverse aortic events in women with Marfan syndrome or with bicuspid aortic valve disease.

## 1. Introduction

Pregnancy induces hemodynamic changes in response to the increased metabolic demands of the mother and fetus [1]. These changes lead to an overall hyperdynamic and hypervolemic cardiovascular state that begins in the first two trimesters and reaches a maximum in the third trimester, during labor and in the postpartum period. The maternal aorta, in particular, is subject to major wall stress and intimal shear forces [2,3]. In addition, pregnancy-induced alterations in estrogen and progesterone levels lead to significant histological changes in the microstructure of the aortic media that cause an increased susceptibility to hemodynamic stress [4]. The aorta reaches a maximal size during the third trimester in pregnant women and stays enlarged by an average of 1 mm at 6 weeks after delivery [5]. In women younger than 40 years, 50% of dissecting aneurysms occur during pregnancy or in the immediate postpartum period [6]. Therefore, many authors consider pregnancy as an important risk factor for adverse aortic events [7,8].

However, most healthy women seem to tolerate physiological changes during gestation without any difficulty [9], whereas women with preexisting aortic disease may actually experience aortic complications [10]. Aortopathies in women of childbearing age have been identified as a significant cause of maternal morbidity and mortality during pregnancy [11]. Due to earlier diagnosis and major advances in medical and surgical therapy, the number of women with genetic aortopathies who are able to undergo pregnancy is continuously rising [12]. Among all hereditary aortopathies, Marfan syndrome constitutes the most frequent cause of adverse aortic outcome during pregnancy [11,13,14]. Some authors have reported an increased risk of aortic events even during long-term follow-up after pregnancy [15,16], as well as of obstetric complications in pregnant women with Marfan syndrome [17,18]. However, most reports are casuistic, and data from larger cohorts remain scarce. 

The lack of conclusive data is even more striking in aortopathy related to bicuspid aortic valve disease. With a prevalence between 0.5% and 1.39% in the general population, the bicuspid aortic valve is the most common congenital cardiovascular malformation [19], yet the risk of pregnancy-related complications in women with bicuspid aortic valve disease has not been sufficiently elucidated [20]. Nonetheless, some pathogenetic similarities between aortopathy in Marfan syndrome and in bicuspid aortic valve disease have led authors to suggest that there is also some increased aortic risk in pregnancy of women with bicuspid aortic valve disease [21,22]. 

However, studies comparing the outcome of pregnancy in Marfan syndrome and in bicuspid aortic valve disease are currently missing. Hence, this retrospective longitudinal, observational study aimed to explore the frequency of pregnancy-associated aortic and obstetric complications among women with Marfan syndrome and with bicuspid aortic valve disease. We provide the first analysis with a special focus on the comparison of immediate and long-term aortic outcomes of pregnancy in women with Marfan syndrome and with bicuspid aortic valve disease.

## 2. Methods

### 2.1. Patients

For our retrospective, observational study we screened clinical records for females aged 18 years or older. We included women with a confirmed diagnosis of Marfan syndrome or bicuspid aortic valve disease. We considered the diagnosis of Marfan syndrome established according to criteria of the revised Ghent nosology. In the absence of a family history of Marfan syndrome, we diagnosed Marfan syndrome with aortic root dilatation combined with ectopia lentis, or with a causative *FBN1* mutation, or with a systemic score ≥ 7 points, or with the combination of ectopia lentis and a *FBN1* mutation known to cause aortic dilatation. When a family history of Marfan syndrome was present, we diagnosed Marfan syndrome with ectopia lentis, or with a systemic score ≥7 points, or with aortic root dilatation (Z-scores ≥2 in case of ages above 20 years or ≥3 below 20 years) [23]. We established presence of bicuspid aortic valve disease with echocardiographic evidence for a congenitally bicuspid aortic valve or a prior event of aortic valve surgery due to congenital bicuspidality. We diagnosed bicuspid aortic valve disease on transthoracic echocardiography with visualization of two versus three aortic valve cusps in systole and diastole in the short-axis view [24]. We excluded all women with a Marfan-like syndrome and a verified genetic mutation other than *FBN1,* and one woman with the coexistence of both Marfan syndrome and bicuspid aortic valve disease. We identified a total of 113 women with a mean age of 40 ± 14 years (range 18–73 years) at first contact and with routine visits to our clinic between December 2003 and March 2017. Of these, 83 were diagnosed as having Marfan syndrome (mean age 39 ± 14 years, range 18–71 years) and 30 had bicuspid aortic valve disease (mean age 41 ± 16 years, range 19–73 years). A causative *FBN1* mutation was present in 79 of 83 women (95%) in the Marfan group. Two individuals with bicuspid aortic valve disease had a mutation in the *NOTCH1* gene (7%). All subjects gave their informed consent before they participated in our study. The study was conducted in accordance with the Declaration of Helsinki, and the protocol was approved by the Ethics Committee of Hamburg (PV5168). We applied STROBE as guideline for study quality [25].

### 2.2. Pregnancy History 

After individual informed consent, we called each woman by telephone and/or sent her a letter to supplement the available data from medical records. We assessed all data by means of a standardized questionnaire. On the basis of questionnaire responses about pregnancy, we categorized all participants as having undergone at least one previous pregnancy as “ever-pregnant”, or as “never-pregnant” without any previous pregnancy. We asked all never-pregnant women to give us their primary reason for not having children (Table 1). We adopted respective response options from Meijboom and colleagues [15,18]. 

### 2.3. Collection of Baseline Data 

We assessed patient charts to record baseline characteristics in all women. Baseline data included age at diagnosis of Marfan syndrome or bicuspid aortic valve disease; age at initial contact to our clinic; body height; body weight; body mass index (BMI); body surface area (BSA) according to Du Bois [26]; serum lipid levels; systolic and diastolic blood pressures; and the intake of anticoagulant agents and/or of aortoprotective medication including beta-adrenergic blockers (BAB), angiotensin-converting enzyme inhibitors (ACEi), or angiotensin-receptor blockers (ARB). We reevaluated imaging data of the entire aorta (CT/MRI) in those patients where annual measurements were performed according to our guideline-based routines, and documented aortic diameters measured at first visit in each woman, if available. We expressed aortic diameters as absolute values and documented diameters for four standard levels of the aorta predefined as follows: aortic root, ascending and descending aorta at the level of the pulmonary trunk, and the suprarenal segment of the abdominal aorta. We obtained aortic diameters only in the native aortic vessel but not in segments replaced by an aortic tube graft. We calculated aortic root Z-scores on the basis of normative data for BSA according to Devereux [27]. Additionally, we assessed syndrome-specific manifestations, including ectopia lentis with the presence of any displacement of the lenses or after surgery for this condition in all women with Marfan syndrome. We also assessed the systemic score in women with Marfan syndrome according to Ghent-2 criteria [23]. We considered a family history of Marfan syndrome or of bicuspid aortic valve disease positive with a first-degree relative having Marfan syndrome or a bicuspid aortic valve, respectively. We also considered a family history positive with a first-degree relative with sudden cardiac death and with preexisting aortic pathology such as aortic aneurysm, previous aortic dissection, or previous aortic surgery. We used standard two-dimensional transthoracic echocardiographic recordings to document any valve dysfunction and its degree (mild, moderate, severe) at baseline contact.

In bicuspid aortic valve disease, we also recorded the type of cusp fusion according to Schaefer as type 1 in case of congenital fusion of the right and left coronary cusp, and type 2 when there was congenital fusion of the right and non-coronary cusp ([28]; Table S1).

#### 2.3.1. Baseline Characteristics in Ever-Pregnant Women According to Diagnosis

First, we analyzed baseline characteristics in 46 ever-pregnant women with Marfan syndrome as compared to 23 ever-pregnant women with bicuspid aortic valve disease (Table 2).

#### 2.3.2. Baseline Characteristics in Women with Marfan Syndrome Or With Bicuspid Aortic Valve Disease According To Pregnancy History

Second, we compared collected baseline data within Marfan group between 46 ever-pregnant and 37 never-pregnant women, and within bicuspid aortic valve disease between 23 ever-pregnant and 7 never-pregnant women (Table S1). 

### 2.4. Obstetric Outcome 

We gathered obstetric data from medical records and questionnaires on pregnancies in ever-pregnant women with Marfan syndrome and in those with bicuspid aortic valve disease. For practical reasons, we documented only the first three pregnancies in eight women with more than three pregnancies at the time of study enrollment. Thereby, we did not show data on 12 pregnancies, among which 5 were in Marfan syndrome and 7 in bicuspid aortic valve disease. We labeled the resulting number of pregnancies with available data as “documented” pregnancies. We termed pregnancies progressing beyond the commonly accepted threshold of fetal viability of 22 weeks of gestation as “completed” pregnancies. Those that did not surpass the threshold were categorized as spontaneous miscarriages, elective abortions, or ectopic pregnancies. In all completed pregnancies, we documented maternal age at delivery, mode of delivery, and the use of anesthesia during labor. We classified delivery according to mode as cesarean section for elective or urgent reasons, and as spontaneous or assisted (by forceps or vacuum) in case of vaginal delivery (Table 3). 

We considered as obstetric complications all obstetric events that occurred during the course of pregnancy, or that took place during or immediately after delivery. Pregnancy-related complications implied pregnancy-induced hypertension (raised blood pressure ≥ 140/90 mmHg after 20 weeks of gestation), preeclampsia (co-occurrence of pregnancy-induced hypertension and proteinuria > 300 mg in a 24 h urine sample or a spot urinary protein to creatinine ratio ≥ 0.3), HELLP syndrome (acronym for the triad of hemolysis, elevated liver enzyme levels, and low platelet account during pregnancy), gestational diabetes, and preterm risk symptoms (cervix insufficiency and/or bleeding from the genital tract). Delivery-related complications included preterm conditions (preterm rupture of membranes defined as spontaneous rupture of membranes in the absence of regular painful contractions, or premature contractions), postpartum hemorrhage (blood loss ≥ 500 mL at vaginal delivery or ≥ 1000 mL at cesarean section), protracted labor, perineal tear or episiotomy, or cardiac/respiratory compromise of any kind during labor ([17]; Table 4). 

### 2.5. Immediate Aortic Outcome of Pregnancy 

We considered any aortic event during pregnancy, delivery, or in the immediate postpartum period (up to 6 months after delivery [7]) as “immediate”. These included dissecting aneurysms and the need for urgent or elective aortic repair (Table 4). As virtually all pregnancies preceded enrollment, we did not have serial echocardiograms of the proximal aorta throughout pregnancy. 

### 2.6. Long-Term Aortic Outcome According To Diagnosis and Pregnancy History 

We considered aortic events as “long-term” with occurrence prior to pregnancy or more than 6 months after delivery. For long-term analysis, we first compared the aortic outcome between ever-pregnant women in both diseases (Table 5 and Table 6). Second, we compared the aortic outcome between ever-pregnant and never-pregnant women with Marfan syndrome, and with bicuspid aortic valve disease, separately (Tables S2, S4 and S6). We defined the following aortic events: (1) aortic dissection or rupture; (2) surgery of the proximal aorta or distal aortic repair, either performed electively due to large or rapidly expanding aneurysm with timing according to guideline-based criteria [29] or carried out urgently with the presence of aortic dissection or rupture; and (3) long-term aortic growth of the four predefined segments of the aorta. We classified aortic dissection according to Stanford as type A with involvement of the ascending aorta and as type B without such involvement [30]. Similarly, we defined surgery of the proximal aorta as any surgery involving the aortic root and ascending aorta, and repair of the distal aorta as endovascular or open repair of the aorta not involving the proximal aortic segment. Proximal aortic surgery comprised aortic root replacement procedures including valve-sparing techniques according to David [31] or to Yacoub [32], as well as composite valve grafting procedures according to Bentall, combining surgery of the aortic root with the replacement of the native aortic valve by a biological or mechanical valve prosthesis [33]. To investigate long-term growth at the predefined aortic levels, we included women only with available imaging data on the native aorta at initial and final contact to our center. 

### 2.7. Statistical Analyses

We compared characteristics between the subgroups according to diagnosis and pregnancy history with the Kruskal–Wallis test for continuous data and the Fisher’s exact test for nominal and categorical data. Unless otherwise specified, we expressed continuous data as means ± standard deviation and categorical data as absolute numbers with respective percentages in parentheses. All tests were performed in an explorative manner.

To investigate the long-term aortic outcome in all women, we performed time-to-event analyses and used the date of birth of each individual as baseline date, and either age at event in case of a present event, or age at final contact in patients without any event during long-term follow-up as key data for time. To quantify the influence of diagnosis and pregnancy history prior to event on the age of aortic dissection, proximal aortic surgery, and distal aortic repair, we used Kaplan–Meier estimators to calculate the cumulative probability of event, with the Log rank to screen for statistical differences between the defined subgroups. In addition, we performed univariate Cox regression analysis and included variables with *p* < 0.05 in a multivariate Cox regression model with forward elimination to identify independent predictors of aortic dissection (Table S3), proximal aortic surgery (Table S5), and distal aortic repair (Table S7) in Marfan syndrome. 

For the analysis of the long-term aortic growth, we displayed time-dependent imaging data by means of spaghetti plots with linear interpolation for each woman separately. Additionally, we compared annual growth (mm/year) at the different aortic levels between ever-pregnant and never-pregnant women with Marfan syndrome (Figures 4–7). Due to the limited availability of imaging data for women with bicuspid aortic valve disease, we presented respective plots only for descriptive means, but neither compared annual growth within this group nor between Marfan syndrome and bicuspid aortic valve disease. Using multiple regression, we quantified the effect of covariates including respective baseline diameters for baseline adjustment, duration of follow-up, diagnosis, and pregnancy history (Table S8).

*p*-values were considered as descriptive measures with values < 0.05 taken to represent indicators of inhomogeneity between groups, where we did not apply methods such as cross validation, bootstrapping, or correction for multiple testing. Statistical tests were performed using IBM-SPSS software (IBM Corp. Released 2013; IBM SPSS Statistics for Windows, Version 22.0. Armonk, NY, USA: IBM Corp), except for the long-term analyses of aortic growth and respective graphics where we used Rstudio (Rstudio Team 2016; Rstudio: Integrated Development for R, Version 1.1.463. Boston, MA: Rstudio, Inc.).

## 3. Results

### 3.1. Pregnancy History 

Women with bicuspid aortic valve disease were ever-pregnant somewhat more often than women with Marfan syndrome (77% vs. 55%; *p* = 0.050). The reasons for not becoming pregnant were heterogeneous, both in never-pregnant women with Marfan syndrome and with bicuspid aortic valve disease (Table 1).

### 3.2. Baseline Characteristics in Ever-Pregnant Women According to Diagnosis

Mean age of ever-pregnant women was similar in Marfan syndrome and bicuspid aortic valve disease, both at initial diagnosis (*p* = 0.775) and at initial contact to our clinic (*p* = 0.980). Use of aortoprotective medication in ever-pregnant females was more common with Marfan syndrome than with bicuspid aortic valve disease (*p* = 0.020). Baseline size of the ascending aorta was larger in ever-pregnant women with bicuspid aortic valve disease than with Marfan syndrome (*p* = 0.002). Conversely, the descending aorta was more dilated in ever-pregnant women with Marfan syndrome than with bicuspid aortic valve disease (*p* = 0.015). A family history of disease (*p*<0.001) or sudden death (*p* = 0.014) was more frequent in Marfan syndrome than in bicuspid aortic valve disease. Mitral valve prolapse was present in 34% of ever-pregnant women with Marfan syndrome, but in none of the women with bicuspid aortic valve disease (*p* = 0.001). In contrast, aortic valve regurgitation (*p* = 0.012), aortic valve stenosis (*p* < 0.001), and coarctation of the aorta (*p* = 0.010) were more common in ever-pregnant women with a bicuspid aortic valve than in those with Marfan syndrome (Table 2).

### 3.3. Baseline Characteristics in Women with Marfan Syndrome or with Bicuspid Aortic Valve Disease According To Pregnancy History

Ever-pregnant women were older at initial contact than never-pregnant women, both in Marfan syndrome (*p* < 0.001) and bicuspid aortic valve disease (*p* = 0.003). Ever-pregnant-women with Marfan syndrome were also older than never-pregnant women when diagnosis was established (*p* = 0.003). In Marfan syndrome, baseline diameters of all aortic segments were larger in ever-pregnant than in never-pregnant women, except for the diameter at the level of the aortic root where the difference was marginal (*p* = 0.060). The distribution of Marfan-specific criteria including systemic score points (*p* = 0.771) and the presence of ectopia lentis (*p* = 0.414) was similar in ever-pregnant and never-pregnant women with Marfan syndrome (Table S1).

### 3.4. Obstetric Outcome 

Of the 148 pregnancies, 93 occurred in 46 ever-pregnant women with Marfan syndrome (63%) and 55 in 23 ever-pregnant women with bicuspid aortic valve disease (37%). However, the number of pregnancies per woman was comparable between both pregnancy groups (*p* = 0.222). Obstetric information was complete in 136 pregnancies (92%). Diagnosis of disease was known before pregnancy in 43% of women with Marfan syndrome as compared to 50% in females with a bicuspid aortic valve (*p* = 0.475). Completed pregnancies (*p* = 0.690), spontaneous miscarriages (*p* = 0.810), elective abortions (*p* = 0.771), and ectopic pregnancies (*p* = 1.000) occurred with similar frequencies in Marfan syndrome and bicuspid aortic valve disease (Table 3).

Hypertensive disorders, including pregnancy-induced hypertension (*p* = 0.139) and preeclampsia, occurred with similar frequencies in both pregnancy groups (*p* = 1.000). The occurrence of delivery-related complications was also comparable between completed pregnancies in Marfan syndrome and in bicuspid aortic valve disease (*p* = 0.112; Table 4). 

### 3.5. Immediate Aortic Outcome of Pregnancy 

In Marfan syndrome, two pregnancies were complicated by aortic dissection (3%), whereas none of the ever-pregnant women with bicuspid aortic valve disease experienced aortic dissection during pregnancy or in the postpartum period (*p* = 0.530; Table 4). 

Case 1—A 30-year-old primigravid woman with known Marfan syndrome and an aortic root diameter of 5.2cm had been closely monitored by echocardiography and received ß-blocker medication during pregnancy. At 35 weeks of gestation, she presented with sudden chest pain during her routine weekly visit to our clinic. An immediate CT scan revealed a type A dissection with involvement of the aortic arch. An urgent cesarean section with subsequent surgery of the proximal aorta and replacement of the aortic arch was successfully performed. 

Case 2—A 32-year-old primigravid woman with known Marfan syndrome and a previously unknown aortic diameter experienced a type A dissection at 38 weeks of gestation. No information on intake of aortoprotective medication were available. The child was delivered by urgent cesarean section followed by the valve-sparing replacement of the proximal aorta. A CT scan was performed 1 week after proximal aortic surgery due to progressive pain and neurological deficits, revealing an extension of the dissection to the aortic bifurcation; thus, a stentgraft was implanted distal of the subclavian artery.

### 3.6. Long-Term Aortic Outcome According To Diagnosis and Pregnancy History 

#### 3.6.1. Long-Term Risk of Aortic Dissection

Aortic dissection occurred in nine ever-pregnant women with Marfan syndrome (21%), whereas none of the ever-pregnant women with bicuspid aortic valve disease experienced such an event in the long term (*p* = 0.022; Table 5). 

**Figure 1:**
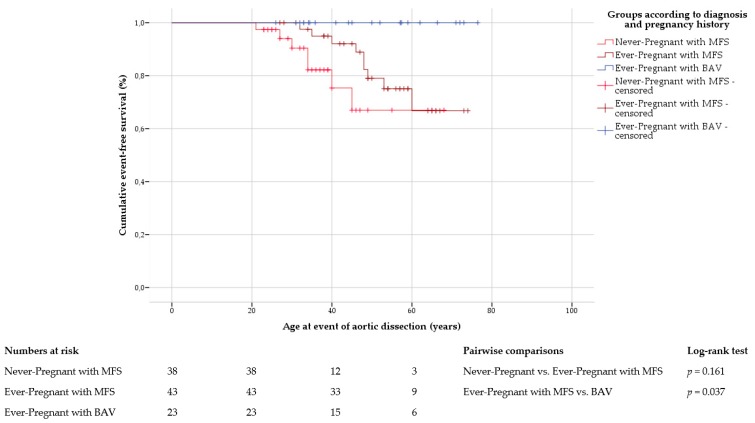
Aortic dissection. Kaplan–Meier curve analysis displays the cumulative probability of freedom from aortic dissection, with comparison between ever-pregnant women with Marfan syndrome (MFS) and with bicuspid aortic valve disease (BAV), and within Marfan group according to pregnancy history. Never-pregnant women with BAV are not shown due to the limited number of cases and lack of events.

Freedom from aortic dissection was significantly lower in ever-pregnant women with Marfan syndrome than in those with bicuspid aortic valve disease (*p* = 0.037; mean freedom from aortic dissection was not calculated because all cases in bicuspid aortic valve disease were censored; Figure 1). 

In Marfan syndrome, the incidence of aortic dissection was similar in ever-pregnant and never-pregnant women (21% vs. 18%; *p* = 1.000; Table S2). Mean freedom from aortic dissection was comparable between ever-pregnant (66 ± 2 years, 95% CI 61–70) and never-pregnant women (57 ± 3 years, 95% CI = 51–64, *p* = 0.161) with Marfan syndrome (Figure 1). There was no association of aortic dissection with previous pregnancy in Marfan women. Instead, multivariate Cox regression analysis identified an elevated systolic blood pressure (hazard ratio (HR) = 1.037; 95% CI 1.010–1.064; *p* = 0.006) and the use of ACEi or ARB medication (HR = 5.211; 95% CI 1.151–23.588; *p* = 0.032) as independent predictors of aortic dissection in Marfan syndrome (Table S3). 

#### 3.6.2. Long-Term Risk of Proximal Aortic Surgery 

Proximal aortic surgery was necessary with a similar proportion in ever-pregnant women with Marfan syndrome and with bicuspid aortic valve disease (*p* = 0.252). However, ever-pregnant women with Marfan syndrome were younger than women with bicuspid aortic valve disease when surgery of the proximal aorta was performed (*p* = 0.041). Although 31% of surgeries in the Marfan group were performed urgently, all surgeries of the proximal aorta in bicuspid aortic valve disease were performed electively (*p* = 0.519; Table 6) and carried out exclusively in ever-pregnant women (Table S4).

**Figure 2:**
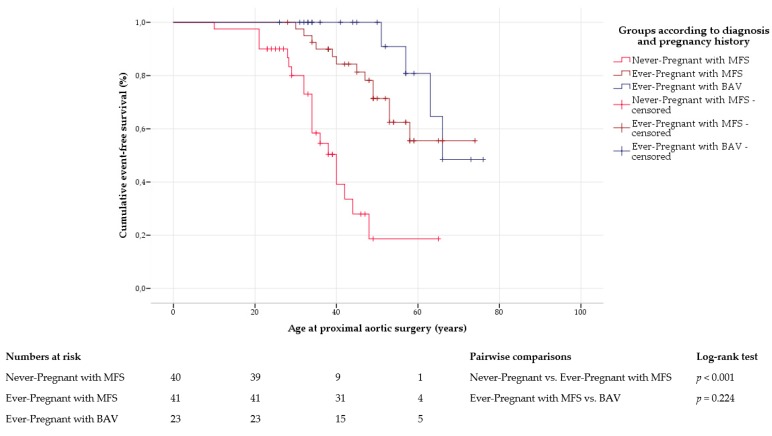
Proximal aortic surgery. Kaplan–Meier curve analysis displays the cumulative probability of proximal aortic surgery, with comparison between ever-pregnant women with Marfan syndrome (MFS) and with bicuspid aortic valve disease (BAV), and within Marfan group according to pregnancy history. Never-pregnant women with BAV are not shown due to the limited number of cases and lack of events.

In Marfan syndrome, proximal aortic surgery was performed with similar frequency in ever-pregnant and never-pregnant women (*p* = 0.116; Table S3). However, Kaplan–Meier curve analysis proved that freedom from proximal aortic surgery was significantly lower in never-pregnant (41 ± 3 years, 95% CI 35–46) than in ever-pregnant women with Marfan syndrome (62 ± 3 years, 95% CI 56–67, *p* < 0.001; Figure 2). Multivariate Cox regression analysis identified the absence of a previous pregnancy at time of proximal aortic surgery (HR = 12.756, 95% CI 1.606–101.304, *p* = 0.016) and a larger diameter of the aortic root (HR = 5.111, 95% CI 1.573–16.602, *p* = 0.007) as independent predictors of proximal aortic surgery in Marfan syndrome (Table S5). 

#### 3.6.3. Long-Term Risk of Distal Aortic Repair 

**Figure 3:**
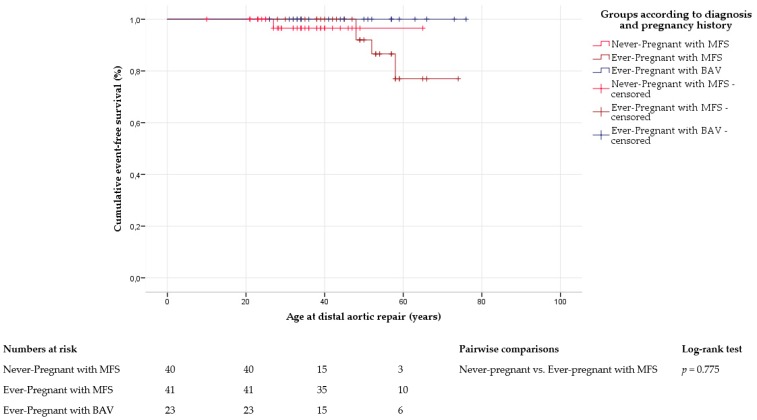
Distal aortic repair. Kaplan–Meier curve analysis displays the cumulative probability of freedom from distal aortic repair, with comparison between ever-pregnant women with Marfan syndrome (MFS) and with bicuspid aortic valve disease (BAV), and within Marfan group according to pregnancy history. Never-pregnant women with BAV are not shown due to the limited number of cases and lack of events.

In Marfan syndrome, distal aortic repair was performed in four ever-pregnant women and one never-pregnant woman (10% vs. 3%; Table S6). Freedom from distal aortic repair was comparable between ever-pregnant (69 ± 2, 95% CI 65–73) and never-pregnant women with Marfan syndrome (64 ± 1, 95% CI 61–66; *p* = 0.775; Figure 3). Cox regression analysis did not identify any independent predictor of distal aortic repair in women with Marfan syndrome (Table S7). In bicuspid aortic valve disease, none of the women underwent distal aortic repair (Table S6). 

#### 3.6.4. Long-Term Aortic Growth

**Figure 4:**
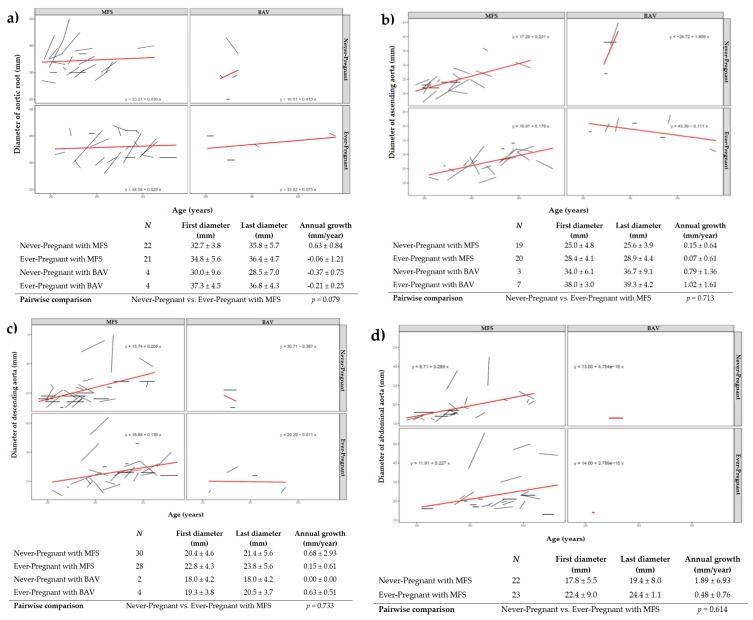
Long-term aortic growth at the level of the aortic root (**a**), ascending aorta (**b**), descending aorta (**c**), and abdominal aorta (**d**) in women with Marfan syndrome (MFS; left panel) and with bicuspid aortic valve disease (BAV; right panel) according to pregnancy history. Red lines visualize slopes of growth for each subgroup. *N* identifies number of individuals with available diameters at first and last contact.

In Marfan syndrome, mean annual growth of the aortic root (*p* = 0.079; Figure 4a) and of all more distal segments of the aorta (Figure 4b–d) was comparable between ever-pregnant and never-pregnant women. Annual aortic growth in women with bicuspid aortic valve is shown in Figure 4a–d. Multiple regression analysis did not find an association of previous pregnancy with increased growth of the aorta in the long term (Table S8).

## 4. Discussion

Our observational study compared the immediate and long-term outcome of pregnancy between women with Marfan syndrome and with bicuspid aortic valve disease. Obstetric outcome in women with Marfan syndrome was comparable to women with bicuspid aortic valve disease. Pregnancy exhibited an increased immediate aortic risk in women with Marfan syndrome, but not in women with bicuspid aortic valve disease. On long-term follow-up, ever-pregnant women with Marfan syndrome were more likely to experience an aortic dissection or to undergo proximal aortic surgery at a younger age than those with bicuspid aortic valve disease. However, pregnancy did not relate to an increased risk of aortic dissection or aortic surgery in the long term, both in Marfan syndrome and in bicuspid aortic valve disease. In Marfan syndrome, pregnancy did not lead to an increased growth of the aorta in the long term.

Pregnancies were less frequent in Marfan syndrome than in bicuspid aortic valve disease. In fact, 29% of never-pregnant women with Marfan syndrome decided to remain childless due to disease-associated fears or a negative advice from their cardiologists. In line with previous findings [15,18], in our study never-pregnant women were initially diagnosed of having Marfan syndrome at a significantly younger age than ever-pregnant women. This may indicate that definitive knowledge of having Marfan syndrome may profoundly affect a woman’s family planning, as was also suggested previously [34]. 

Aortic root diameters and Z-scores at baseline were comparable between ever-pregnant women with Marfan syndrome and with bicuspid aortic valve disease, and also between ever-pregnant and never-pregnant females in both diagnosis groups, respectively. Several studies corroborated the fact that there is no association between previous pregnancy and the size of the proximal aorta in women with Marfan syndrome [15,35,36] or with bicuspid aortic valve disease [22]. Diameters of the ascending segment of the aorta were larger in ever-pregnant women with bicuspid aortic valve disease than with Marfan syndrome, whereas more distal segments of the aorta were more dilated in ever-pregnant women with Marfan syndrome than with bicuspid aortic valve disease. Previous investigations confirmed that the anatomic site of major aortic pathology was distinct in both entities and showed that the maximal aortic dilatation occurred at the ascending aorta in individuals with bicuspid aortic valve disease [37,38]. In accordance with the literature, we found coarctation of the aorta only with bicuspid aortic valve disease but not with Marfan syndrome [39], and identified mitral valve prolapse only with Marfan syndrome but not with bicuspid aortic valve disease [40].

Delivery-related complications were somewhat more frequent in Marfan syndrome (37%) than in bicuspid aortic valve disease (21%), although the difference was not significant. Other studies on pregnancy in Marfan syndrome corroborated rates of obstetric complications, which were comparable to our findings [18,36]. Study data on the obstetric outcome of women are available for Marfan syndrome but not for bicuspid aortic valve disease. 

Two women with Marfan syndrome experienced aortic dissection during a total of 62 completed pregnancies, accounting for a rate of 3% in our study. A literature review that comprised 12 studies (39 aortic dissections among 1271 pregnancies in 832 women with Marfan syndrome) yielded a similar composite risk rate of approximately 3.1% [41]. Conversely, our study identified no aortic dissection during a total of 36 completed pregnancies in 23 ever-pregnant women with bicuspid aortic valve disease. In line with current European guidelines, our findings suggest that pregnancy in bicuspid aortic valve disease carries a low immediate risk for the maternal aorta [1].

In the long-term follow-up, our study demonstrated a higher risk of aortic dissection in ever-pregnant with Marfan syndrome than in women with a congenital bicuspid aortic valve, who remained free of aortic dissection events. This finding reflects the higher long-term aortic risk exhibited by Marfan aortopathy than by aortopathy related to bicuspid aortic valve disease [14,42]. In Marfan syndrome, we observed a similar proportion of dissecting aneurysms in ever-pregnant (21%) and never-pregnant women (18%). However, findings on pregnancy-related long-term risk of aortic dissection varied across previous studies—although some studies did not find an association of aortic dissection with previous pregnancy in Marfan syndrome [43], in some other investigations ever-pregnant women with Marfan syndrome were more likely to have experienced an aortic dissection than never-pregnant women [16,35]. Hypertension is a well-known risk factor of aortic dissection [44] and was also identified as an independent predictor of aortic dissection among Marfan patients in our study. Interestingly, the use of ACEi/ARB medication was associated with aortic dissection in the long term. However, systolic blood pressure was similar in women with Marfan syndrome irrespective of ACEi/ARB medication. An insufficient blood-lowering effect and/or reduced drug compliance may thus be a possible explanation for our somewhat unexpected finding. Our finding may also reflect the more common use of ACEi/ARB medication among Marfan patients with high-risk aortic pathology. 

The frequency of proximal aortic surgery was comparable in ever-pregnant women with Marfan syndrome and with bicuspid aortic valve disease. However, ever-pregnant women with Marfan syndrome underwent surgery of the proximal aorta at a significantly younger age. Irrespective of pregnancy history, previous studies confirmed that aortic surgery was necessary at an earlier age in Marfan syndrome than in bicuspid aortic valve disease [42]. In our study, proximal artic surgery was performed with similar frequency in ever-pregnant and never-pregnant women with Marfan syndrome. Although some studies suggested that pregnancy may increase the long-term risk of proximal aortic surgery in women with Marfan syndrome [16,35], in our study, never-pregnant women with Marfan syndrome were younger when surgery was performed. Our findings show that women with a more severe involvement of the aorta and the consecutive need for proximal aortic surgery at a young age were likely to decide against pregnancy due to the associated risks. In Marfan syndrome, growth at all aortic levels was comparable between ever-pregnant and never-pregnant women. Previous studies also detected similar long-term growth of the aortic root in both Marfan subgroups [15,36]. However, comparable imaging data on more distal aortic segments are missing in the current literature. 

As we performed a retrospective study in a survival-only cohort, no information on fatal cardiovascular complications were available. Given the retrospective design of our study, missing or incomplete data were inevitable. The sample size of our study was limited, and statistical analyses therefore have to be considered with caution. The retrospective nature of our study also accounted for the lack of data on peripartum aortic diameters. 

Large multicenter studies, ideally of prospective design, may be necessary to further elucidate and compare the immediate and long-term impact of pregnancy on the aortic outcome in women with Marfan syndrome and with bicuspid aortic valve disease.

## Figures and Tables

**Table 1:** Pregnancy history of women with Marfan syndrome (MFS) and with bicuspid aortic valve (BAV).

Variable	MFS	BAV	*p*
Number of individuals	83	30	
**Ever-pregnant women**	46 (55%)	23 (77%)	0.050
Women with one pregnancy	18 (22%)	6 (20%)	1.000
Women with two pregnancies	13 (16%)	9 (30%)	0.109
Women with at least three pregnancies	15 (18%)	8 (27%)	0.427
**Never-pregnant women**	37 (45%)	7 (23%)	
*Primary reasons for not having children*			
Voluntarily childless	13 (35%)	4 (57%)	
Young age and/or no partner	7 (19%)	3 (43%)	
Fertility problems	3 (8%)	0	
Disease-associated fears	9 (24%)	0	
Negative advice from cardiologist	2 (5%)	0	
Thrombophilia	1 (3%)	0	
Unknown	2 (5%)	0	

**Table 2:** Baseline characteristics in ever-pregnant women with Marfan syndrome (MFS) and with bicuspid aortic valve disease (BAV).

Variable	Ever-Pregnant with MFS	Ever-Pregnant with BAV	*p*
Number of individuals	46	23	
Age at diagnosis (years)	35 ± 13	36 ± 24	0.775
Age at initial contact (years)	45 ± 12	46 ± 12	0.980
Body height (cm)	179 ± 8	166 ± 8	<0.001
Body weight (kg)	78 ± 18	67 ± 14	0.002
BMI (kg/m²)	24 ± 6	24 ± 4	0.970
BSA (m²)	2.0 ± 0.2	1.7 ± 0.2	<0.001
Total cholesterol (mg/dL)	199 ± 40	202 ± 40	0.738
HDL cholesterol (mg/dL)	63 ± 17	71 ± 21	0.083
LDL cholesterol (mg/dL)	109 ± 36	107 ± 29	0.923
Systolic blood pressure (mm Hg)	128 ± 19	128 ± 23	0.945
Diastolic blood pressure (mm Hg)	76 ± 11	79 ± 11	0.249
BAB medication	25 (54%)	12 (52%)	1.000
ACEi or ARB medication	24 (52%)	5 (22%)	0.020
Anticoagulation	7 (15%)	4 (17%)	1.000
Aortic sinus diameter (cm) ^1^	3.7 ± 0.7	3.3 ± 0.6	0.360
Aortic sinus Z-score ^1^	1.8 ± 2.8	1.0 ± 2.3	0.635
Diameter of ascending aorta (cm) ^1^	2.9 ± 0.6	3.7 ± 0.9	0.002
Diameter of descending aorta (cm) ^1^	2.6 ± 1.1	2.0 ± 0.5	0.015
Diameter of abdominal aorta (cm) ^1^	2.3 ± 0.9	1.8 ± 0.3	0.098
Family history of disease	33 (72%)	2 (9%)	<0.001
Family history of sudden death	21 (47%)	3 (14%)	0.014
At least moderate degree of MVR	3/43 (7%)	1 (4%)	1.000
MV prolapse	15/44 (34%)	0	0.001
At least moderate degree of AVR	3/43 (12%)	8 (35%)	0.012
At least moderate degree of AVS	0	13 (57%)	<0.001
Coarctation of the aorta	0	4 (17%)	0.010
At least moderate degree of TVR	2/43 (5%)	0	0.539

ACEi, angiotensin-converting enzyme inhibitors; ARB, angiotensin-receptor blockers; AVR, aortic valve regurgitation; AVS, aortic valve stenosis; BAB, beta-adrenergic blockers; BMI, body mass index; BSA, body surface area; HDL, high-density lipoprotein; LDL, low-density lipoprotein; MV, mitral valve; MVR, mitral valve regurgitation; TVR, tricuspid valve regurgitation. If less than total, we presented the number of individuals with available information behind a slash. ^1^ Diameters of aortic segments were obtained at initial presentation only in those with native vessels at the time of measurement.

**Table 3:** General obstetric data in ever-pregnant women with Marfan syndrome (MFS) and with bicuspid aortic valve disease (BAV).

Variable	Pregnancies in MFS	Pregnancies in BAV	*p*
Number of women	46	23	0.050
Total number of pregnancies	93	55	
Pregnancies per woman	2.0 ± 1.1	2.4 ± 1.4	0.222
**Number of documented pregnancies**	88 (95%)	48 (87%)	
Diagnosis of MFS or BAV before pregnancy	38 (43%)	24 (50%)	0.475
Spontaneous miscarriage	15/87 (17%)	7 (15%)	0.810
Elective abortion	9/87 (10%)	4 (8%)	0.771
Ectopic pregnancy	1/87 (1%)	1 (2%)	1.000
**Number of completed pregnancies**	62 (70%)	36 (75%)	0.690
Maternal age at delivery (years)	28 ± 5	28 ± 5	0.938
**Cesarean section**	19/62 (31%)	16/36 (44%)	0.194
*Elective section*	14 (23%)	13 (36%)	0.166
Cardiac reason	5 (8%)	3 (8%)	1.000
Fetal reason	2 (3%)	3 (8%)	0.353
Obstetric reason	7 (11%)	7 (19%)	0.370
*Urgent section*	5 (8%)	3 (8%)	1.000
Cardiac reason	4 (7%)	0	0.293
Fetal reason	0	2 (6%)	0.133
Obstetric reason	1 (2%)	1 (3%)	1.000
**Vaginal delivery**	43/62 (69%)	20/36 (56%)	0.194
Spontaneous	37 (60%)	18 (50%)	0.402
Assisted (forceps or vacuum)	6 (10%)	2 (6%)	0.706
**Anesthesia**	25/61 (41%)	20/35 (57%)	0.142
Regional	16 (26%)	12 (34%)	0.486
General	9 (15%)	8 (23%)	0.406

If numbers of pregnancies with information on a respective variable were less than the total, we presented the number of pregnancies with the available information behind a slash.

**Table 4:** Obstetric and aortic outcome of completed pregnancies in ever-pregnant women with Marfan syndrome (MFS) and with bicuspid aortic valve disease (BAV).

Variable	Pregnancies in MFS	Pregnancies in BAV	*p*
Number of completed pregnancies	62	36	
**Pregnancy-related complication**	16 (26%)	13 (36%)	0.359
Pregnancy-induced hypertension	1 (2%)	3 (8%)	0.139
Preeclampsia and/or HELLP syndrome	3 (5%)	1 (3%)	1.000
Gestational diabetes	0	1 (3%)	0.367
Aortic dissection	2 (3%)	0	0.530
Preterm risk symptom	7 (11%)	6 (17%)	0.540
Other ^1^	3 (5%)	2 (6%)	1.000
**Delivery-related complication**	23 (37%)	7 (21%)	0.112
Preterm condition	6 (10%)	2 (6%)	0.708
Postpartum hemorrhage	6 (10%)	2 (6%)	0.708
Protracted labor	3 (5%)	1 (3%)	1.000
Perineal tear or episiotomy	5 (8%)	1 (3%)	0.418
Cardiac/respiratory compromise	3 (5%)	1 (3%)	1.000

^1^ Other pregnancy-related complications included hydronephrosis with/without urosepsis, intestinal prolapse, and pregnancy-associated allergy.

**Table 5:** Aortic dissection in ever-pregnant women with Marfan syndrome (MFS) and with bicuspid aortic valve disease (BAV).

Outcome Variable	Ever-Pregnant with MFS ^1^	Ever-Pregnant with BAV	*p*
Number of individuals ^2^	43	23	
Age at final contact (years)	52 ± 12	50 ± 16	0.484
Number of dissections	9 (21%)	0	0.022
Dissection by age (years)	46 ± 9		
*Type according to Stanford*			
Type A	4 (44%)	0	
Type B	5 (56%)	0	

^1^ Two women in the MFS cohort with aortic dissection during pregnancy were excluded from long-term analysis. ^2^ One ever-pregnant woman with MFS had her first pregnancy after aortic dissection and was therefore categorized as being never-pregnant (see Table S2).

**Table 6:** Proximal aortic surgery in ever-pregnant women with Marfan syndrome (MFS) and with bicuspid aortic valve disease (BAV).

Outcome Variable	Ever-Pregnant with MFS ^1^	Ever-Pregnant with BAV	*p*
Number of individuals ^2^	41	23	
Age at final contact (years)	53 ± 12	50 ± 16	0.345
Number of proximal surgeries	13 (32%)	4 (17%)	0.252
Proximal surgery by age (years)	44 ± 9	59 ± 7	0.041
*Proximal surgery by indication*			
Prophylactic surgery	9 (69%)	4 (100%)	0.519
Urgent surgery	4 (31%)	0	
(rupture/dissection)			
*Proximal surgery by technique*			
Valve-sparing procedures	8 (62%)	1 (25%)	
Aortic root replacement	2 (15%)	2 (50%)	
(biological valve)			
Aortic root replacement	2 (15%)	1 (25%)	
(mechanical valve)			
Other	1 (8%)	0	

^1^ Two women in the MFS cohort with aortic dissection during pregnancy were excluded from long-term analysis. ^2^ Three women with MFS had their first pregnancies after proximal aortic surgery had been performed and were therefore categorized as being never-pregnant (see Table S4).

## Supplementary Materials

The following are available online at https://www.mdpi.com/2077-0383/9/4/1124/s1, Table S1: Baseline characteristics in women with Marfan syndrome (MFS) and with bicuspid aortic valve disease (BAV) according to pregnancy history. Table S2: Aortic dissection in women with Marfan syndrome (MFS) and with bicuspid aortic valve disease (BAV) according to pregnancy history prior to event. Table S3: Aortic dissection in 81 individuals with Marfan syndrome (MFS). Table S4: Proximal aortic surgery in women with Marfan syndrome (MFS) and with bicuspid aortic valve disease (BAV) according to pregnancy history prior to event. Table S5: Proximal aortic surgery in 81 individuals with Marfan syndrome (MFS). Table S6: Distal aortic repair in women with Marfan syndrome (MFS) and with bicuspid aortic valve disease (BAV) according to pregnancy history prior to event. Table S7: Distal aortic repair in 81 individuals with Marfan syndrome (MFS). Table S8: Long-term aortic growth in individuals with Marfan syndrome (MFS) and with bicuspid aortic valve disease (BAV).
