# Supplementary material for: Marfan Syndrome Versus Bicuspid Aortic Valve Disease: Comparative Analysis of Obstetric Outcome and Pregnancy-Associated Immediate and Long-Term Aortic Complications
Source: J Clin Med. 2020 Apr 15;9(4):1124. doi: 10.3390/jcm9041124 (PMC7230569; doi:10.3390/jcm9041124)
Supplement: Supplementary file 1 [file jcm-09-01124-s001.pdf]

Supplementary Materials: Marfan Syndrome Versus Bicuspid Aortic Valve Disease: Comparative Analysis Of Obstetric Outcome And Pregnancy-Associated Immediate And Long-Term Aortic Complications

Betül Toprak, Katalin Szöcs, Elvin Zengin-Sahm, Christoph Sinning, Amra Hot, Peter Bannas, Kurt Hecher, Bernd Hüneke, Thomas S. Mir, Meike Rybczynski, Evaldas Girdauskas, Stefan Blankenberg and Yskert von Kodolitsch

Table S1. Baseline characteristics in women with Marfan syndrome (MFS) and with bicuspid aortic valve disease (BAV) according to pregnancy history.

Variable	MFS (*N* = 83)			BAV (*N* = 30)		
	**Ever-Pregnant**	**Never-Pregnant**	***p***	**Ever-Pregnant**	**Never-Pregnant**	***p***
Number of individuals	46	37		23	7	
Age at initial contact (years)	45 ± 12	32 ± 12	<0.001	46 ± 16	27 ± 3	0.003
Age at diagnosis (years)	35 ± 13	20 ± 15	<0.001	36 ± 24	19 ± 10	0.111
Body height (cm)	179 ± 8	180 ± 7	0.177	166 ± 8	171 ± 7	0.169
Body weight (kg)	78 ± 18	70 ± 12	0.024	67 ± 14	63 ± 9	0.712
BMI (kg/m^2)	24 ± 6	22 ± 3	0.008	24 ± 4	22 ± 3	0.135
BSA (m^2)	2.0 ± 0.2	1.9 ± 0.2	0.084	1.7 ± 0.2	1.7 ± 0.1	0.941
Total cholesterol (mg/dl)	199 ± 40	180 ± 28	0.048	202 ± 40	164 ± 22	0.032
HDL cholesterol (mg/dl)	63 ± 17	60 ± 15	0.453	71 ± 21	61 ± 13	0.130
LDL cholesterol (mg/dl)	109 ± 36	95 ± 24	0.055	107 ± 29	86 ± 22	0.095
Systolic blood pressure (mm Hg)	128 ± 19	125 ± 18	0.611	128 ± 23	122 ± 4	0.622
Diastolic blood pressure (mm Hg)	76 ± 11	72 ± 11	0.163	79 ± 11	75 ± 4	0.312
BAB medication	25 (54%)	19 (51%)	0.786	12 (52%)	1 (14%)	0.104
ACEi or ARB medication	24 (52%)	14 (38%)	0.193	5 (22%)	0	0.304
Anticoagulation	7 (15%)	6 (16%)	0.901	4 (17%)	1 (14%)	1.000
Aortic root diameter (cm)[1]	3.7 ± 0.7	3.3 ± 0.4	0.060	3.3 ± 0.6	3.0 ± 0.7	0.243
Aortic root Z-score (Devereux)[1]	1.8 ± 2.8	1.1 ± 1.6	0.692	1.0 ± 2.3	0.2 ± 2.6	0.289
Diameter of ascending aorta (cm)[1]	2.9 ± 0.6	2.7 ± 1.4	0.002	3.7 ± 0.9	3.2 ± 0.5	0.061
Diameter of descending aorta (cm)[1]	2.6 ± 1.1	2.0 ± 0.5	0.001	2.0 ± 0.5	1.8 ± 0.3	0.550
Diameter of abdominal aorta (cm)[1]	2.3 ± 0.9	1.7 ± 0.5	0.002	1.8 ± 0.3	1.4 ± 0.2	0.074
Ghent-2 systemic score (points)	6.7 ± 3.2	6.9 ± 3.3	0.771			
Ectopia lentis	23 (53%)	15 (44%)	0.414			
Family history of disease	33 (72%)	19 (51%)	0.056	2 (9%)	0	1.000
Family history of sudden death	21 (47%)	17 (56%)	0.948	3 (14%)	1 (14%)	1.000

At least moderate degree of MVR	3/43 (7%)	6/35 (17%)	0.285	1 (4%)	0	1.000
MV prolapse	15/44 (34%)	20/36 (56%)	0.054	0	0	
At least moderate degree of AVR	3/43 (12%)	4/34 (7%)	0.693	8 (35%)	2 (29%)	1.000
At least moderate degree of AVS	0	0		13 (57%)	3 (43%)	0.675
BAV fusion type 1				11/12 (92%)	5/5 (100%)	1.000
BAV fusion type 2				1/12 (8%)		
Coarctation of the aorta	0	0		4 (17%)	0	0.548
At least moderate degree of TVR	2/43 (5%)	1/34 (3%)	1.000	0	0	

ACEi, angiotensin-converting enzyme inhibitors; ARB, angiotensin receptor blockers; AVR, aortic valve regurgitation; AVS, aortic valve stenosis; BAB, beta-adrenergic blockers; BMI, body mass index; BSA, body surface area; HDL, high-density lipoprotein; LDL, low-density lipoprotein; MV, mitral valve; MVP, mitral valve prolapse; MVR, mitral valve regurgitation; *N*, number of individuals; TVR, tricuspid valve regurgitation. If less than total, we present the number of individuals with available information behind a slash. [1] Diameters of aortic segments were obtained at initial presentation only in those with native vessels at time of measurement.

Table S2. Aortic dissection in women with Marfan syndrome (MFS) and with bicuspid aortic valve disease (BAV) according to pregnancy history prior to event.

Outcome Variable	MFS (*N* = 81)[1]			BAV (*N* = 30)		
	**Ever-Pregnant**	**Never-Pregnant**	***p***	**Ever-Pregnant**	**Never-Pregnant**	***p***
Number of individuals[2]	43	38		23	7	
Age at final contact (years)	52 ± 12	38 ± 12	<0.001	50 ± 16	30 ± 4	0.002
Number of dissections	9 (21%)	7 (18%)	1.000	0	0	
Dissection by age (years)	46 ± 9	33 ± 8	0.011			
Type according to Stanford						
Type A	4 (44%)	3 (43%)	1.000	0	0	
Type B	5 (56%)	4 (57%)		0	0	

N identifies number of individuals. [1] Two women in the MFS group with aortic dissection during pregnancy were excluded from long-term analysis. [2] One ever-pregnant individual with MFS had no pregnancy prior to event of dissection and was therefore categorized as being never-pregnant.

Table S3. Aortic dissection in 81 individuals with Marfan syndrome (MFS)[1].

	**Aortic Dissection**		**Univariate Cox Regression Analysis**			
**Variable**	**Absent (*N* = 65)**	**Present (*N* = 16)**	**Hazard Ratio**	**Lower 95% CI**	**Upper 95% CI**	***p***
Age at diagnosis (years)	28 ± 17	31 ± 13	0.979	0.948	1.012	0.212
Previous pregnancy	34 (52%)	9 (56%)	0.491	0.177	1.358	0.171
BMI (kg/m^2)	23 ± 4	25 ± 7	1.035	0.951	1.127	0.429
BSA (m^2)	1.9 ± 0.2	2.0 ± 0.2	4.279	0.446	41.046	0.208
Total cholesterol (mg/dl)	190 ± 38	193 ± 32	0.994	0.981	1.008	0.414
HDL cholesterol (mg/dl)	62 ± 16	59 ± 19	0.985	0.954	1.016	0.341

LDL cholesterol (mg/dl)	104 ± 33	100 ± 27	0.992	0.978	1.007	0.289
Systolic blood pressure (mm Hg)	123 ± 16	144 ± 20	1.036	1.011	1.062	0.005
Diastolic blood pressure (mm Hg)	74 ± 11	78 ± 11	1.016	0.969	1.065	0.509
BAB medication	29 (45%)	14 (88%)	4.411	0.998	19.491	0.050
ACEi or ARB medication	24 (37%)	13 (81%)	4.805	1.367	16.889	0.014
Ghent-2 systemic score (points)	6.8 ± 3.2	6.5 ± 3.2	0.986	0.850	1.145	0.857
Family history of disease	43 (66%)	8 (50%)	0.670	0.247	1.812	0.430
Family history of sudden death	27 (42%)	10 (63%)	1.656	0.600	4.566	0.330
Family history of aortopathy	27 (42%)	7 (44%)	1.404	0.519	3.800	0.504
MV regurgitation	31/61 (51%)	11/15 (73%)	2.219	0.700	7.033	0.176
MV prolapse	30/63 (48%)	4/15 (27%)	0.580	0.184	1.825	0.351
AV regurgitation	17/61 (28%)	8/15 (53%)	1.758	0.637	4.856	0.276
TV regurgitation	46/61 (75%)	10/15 (67%)	1.047	0.356	3.078	0.933
**Prediction of Aortic Dissection**			**Multivariate Cox Regression Analysis**			
			**Hazard Ratio**	**Lower 95% CI**	**Upper 95% CI**	***p***
Systolic blood pressure (mm Hg)			1.037	1.010	1.064	0.006
ACEi or ARB medication			5.211	1.151	23.588	0.032

ACEi, angiotensin-converting enzyme inhibitors; ARB, angiotensin receptor blockers; AV, aortic valve; BAB, beta-adrenergic blockers; BMI, body mass index; BSA, body surface area; CI, confidence interval; HDL, high-density lipoprotein; LDL, low-density lipoprotein; MV, mitral valve; *N*, number of events; TV, tricuspid valve. If less than total, we present the number of individuals with available information behind a slash. [1] Two women in the MFS cohort with aortic dissection during pregnancy were excluded from long-term analysis.

Table S4. Proximal aortic surgery in women with Marfan syndrome (MFS) and with bicuspid aortic valve disease (BAV) according to pregnancy history prior to event.

**Outcome Variable**	**MFS (*N* = 81)**[1]			**BAV (*N* = 30)**		
	**Ever-Pregnant**	**Never-Pregnant**	***p***	**Ever-Pregnant**	**Never-Pregnant**	***p***
Number of individuals[2]	41	40		23	7	
Age at final contact (years)	53 ± 12	38 ± 12	<0.001	50 ± 16	30 ± 4	0.002
Number of proximal surgeries	13 (32%)	20 (50%)	0.116	4 (17%)	0	0.548
Proximal surgery by age (years)	44 ± 9	32 ± 9	0.001	59 ± 7		
Proximal surgery by indication						
Prophylactic surgery	9 (69%)	17 (85%)	0.393	4 (100%)	0	0.548
Urgent surgery (rupture/dissection)	4 (31%)	3 (15%)		0	0	
Proximal surgery by technique						
Valve-sparing procedures	8 (62%)	16 (80%)		1 (25%)	0	

Aortic root replacement (biological valve)	2 (15%)	0	2 (50%)	0
Aortic root replacement (mechanical valve)	2 (15%)	2 (10%)	1 (25%)	0
Other	1 (8%)	2 (10%)	0	0

N identifies number of individuals. [1] Two women in the MFS cohort with aortic dissection during pregnancy were excluded from long-term analysis. [2] Three women with MFS had their first pregnancies after proximal aortic surgery had been performed and were therefore categorized as being never-pregnant.

Table S5. Proximal aortic surgery in 81 individuals with Marfan syndrome (MFS)[1].

	**Proximal Aortic Surgery**		**Univariate Cox Regression Analysis**			
**Variable**	**Absent (*N* = 48)**	**Present (*N* = 33)**	**Hazard Ratio**	**Lower 95% CI**	**Upper 95% CI**	***p***
Age at diagnosis (years)	28 ± 17	30 ± 15	1.007	0.985	1.029	0.555
Previous pregnancy	28 (58%)	13 (39%)	0.203	0.096	0.431	<0.001
BMI (kg/m^2)	23 ± 4	24 ± 5	1.028	0.971	1.088	0.341
BSA (m^2)	1.9 ± 0.2	1.9 ± 0.2	1.736	0.348	8.654	0.501
Total cholesterol (mg/dl)	194 ± 37	185 ± 36	0.996	0.986	1.006	0.394
HDL cholesterol (mg/dl)	61 ± 16	61 ± 14	1.002	0.980	1.024	0.876
LDL cholesterol (mg/dl)	107 ± 33	98 ± 28	0.995	0.983	1.006	0.352
Systolic blood pressure (mm Hg)	127 ± 16	127 ± 21	1.003	0.985	1.021	0.767
Diastolic blood pressure (mm Hg)	74 ± 10	75 ± 12	1.011	0.978	1.046	0.508
BAB medication	26 (54%)	17 (52%)	0.993	0.501	1.970	0.984
ACEi or ARB medication	23 (48%)	14 (42%)	0.855	0.428	1.706	0.657
Aortic root diameter (cm)	3.4 ± 0.6	4.1 ± 0.5	2.544	1.072	6.035	0.034
Aortic root Z-score	1.0 ± 2.1	4.3 ± 1.6	1.355	1.087	1.690	0.007
Diameter of ascending aorta (cm)	2.8 ± 1.1	3.1 ± 0.9	1.274	0.477	3.403	0.629
Diameter of descending aorta (cm)	2.2 ± 1.1	2.6 ± 0.6	1.065	0.779	1.455	0.694
Diameter of abdominal aorta (cm)	2.0 ± 0.8	2.2 ± 0.7	0.968	0.586	1.597	0.898
Ghent-2 systemic score (points)	6.8 ± 3.3	6.7 ± 3.1	1.000	0.896	1.116	0.998
Family history of disease	27 (56%)	24 (73%)	1.488	0.690	3.211	0.311
Family history of sudden death	20 (43%)	17 (52%)	1.281	0.645	2.544	0.479
Family history of aortopathy	21 (44%)	12 (36%)	0.713	0.350	1.451	0.350
MV regurgitation	28 (60%)	13 (45%)	0.624	0.300	1.300	0.208
MV prolapse	23 (49%)	11 (36%)	0.642	0.307	1.343	0.239
AV regurgitation	19 (40%)	7 (24%)	0.632	0.270	1.481	0.291
TV regurgitation	38 (81%)	18 (62%)	0.631	0.298	1.339	0.230

**Prediction of Proximal Aortic Surgery**	**Multivariate Cox Regression Analysis**			
	**Hazard Ratio**	**Lower 95% CI**	**Upper 95% CI**	***p***

Absence of previous pregnancy	12.756	1.606	101.304	0.016
Aortic root diameter (cm)	5.111	1.573	16.602	0.007

ACEi, angiotensin-converting enzyme inhibitors; ARB, angiotensin receptor blockers; AV, aortic valve; BAB, beta-adrenergic blockers; BMI, body mass index; BSA, body surface area; CI, confidence interval; HDL, high-density lipoprotein; LDL, low-density lipoprotein; MV, mitral valve; *N*, number of events; TV, tricuspid valve. If less than total, we present the number of individuals with available information behind a slash. [1] Two women in the MFS cohort with aortic dissection during pregnancy were excluded from long-term analysis.

Table S6. Distal aortic repair in women with Marfan syndrome (MFS) and with bicuspid aortic valve disease (BAV) according to pregnancy history prior to event.

Outcome Variable	MFS (*N* = 81)[1]			BAV (*N* = 30)		
	**Ever-Pregnant**	**Never-Pregnant**	***p***	**Ever-Pregnant**	**Never-Pregnant**	***p***
Number of individuals	44	37		23	7	
Age at final contact (years)	52 ± 12	38 ± 12	<0.001	50 ± 16	30 ± 4	0.002
Number of distal repairs	4 (10%)	1 (3%)	0.359	0	0	
Distal repair by age (years)	52 ± 5	27				
Distal repair by indication						
Elective procedure (true or false lumen expansion)	2 (50%)	0	1.000	0	0	
Urgent procedure (rupture/dissection)	2 (50%)	1 (100%)		0	0	
Distal repair by technique						
Surgical	2 (50%)	0	1.000	0	0	
Endovascular	2 (50%)	1 (100%)		0	0	

N identifies number of events. [1] Two women in the MFS group with aortic dissection during pregnancy were excluded from long-term analysis.

Table S7. Distal aortic repair in 81 individuals with Marfan syndrome (MFS)[1].

Variable	Distal Aortic Repair		Univariate Cox Regression Analysis			
	**Absent (*N* = 76)**	**Present (*N* = 5)**	**Hazard Ratio**	**Lower 95% CI**	**Upper 95% CI**	***p***
Age at diagnosis (years)	28 ± 16	36 ± 15	1.025	0.968	1.086	0.401
Previous pregnancy	37 (49%)	4 (80%)	1.269	0.134	12.016	0.835
BMI (kg/m^2)	23 ± 5	24 ± 3	1.030	0.895	1.185	0.678
BSA (m^2)	1.9 ± 0.2	2.0 ± 0.1	3.517	0.059	210.481	0.547
Total cholesterol (mg/dl)	189 ± 37	219 ± 22	1.021	0.997	1.045	0.091
HDL cholesterol (mg/dl)	60 ± 15	67 ± 20	1.031	0.972	1.094	0.310
LDL cholesterol (mg/dl)	102 ± 31	125 ± 30	1.019	0.994	1.044	0.131
Systolic blood pressure (mm Hg)	127 ± 19	131 ± 6	1.009	0.966	1.054	0.684
Diastolic blood pressure (mm Hg)	74 ± 11	74 ± 7	0.997	0.908	1.095	0.951

BAB medication	40 (53%)	3 (60%)	1.316	0.219	7.901	0.764
ACEi or ARB medication	36 (47%)	1 (20%)	0.329	0.037	2.945	0.320
Aortic root diameter (cm)	3.4 ± 0.5	4.7 ± 1.3	16.081	0.666	388.252	0.087
Aortic root Z-score	1.3 ± 2.1	5.6 ± 5.2	2.051	0.890	4.731	0.092
Diameter of ascending aorta (cm)	2.7 ± 0.6	4.9 ± 2.8	2.820	1.169	6.801	0.021
Diameter of descending aorta (cm)	2.2 ± 0.6	4.3 ± 3.6	2.305	1.017	5.221	0.045
Diameter of abdominal aorta (cm)	2.0 ± 0.6	4.2 ± 0.6	5.734	1.395	23.563	0.015
Ghent-2 systemic score (points)	6.6 ± 3.2	9.0 ± 3.2	1.258	0.928	1.704	0.140
Family history of disease	49 (65%)	2 (40%)	0.344	0.057	2.062	0.243
Family history of sudden death	35 (47%)	2 (40%)	0.699	0.116	4.207	0.696
Family history of aortopathy	31 (41%)	2 (40%)	0.948	0.158	5.682	0.954
MV regurgitation	37 (52%)	4 (80%)	2.980	0.332	26.713	0.329
MV prolapse	33 (45%)	1 (20%)	0.302	0.034	2.713	0.285
AV regurgitation	23 (32%)	3 (60%)	3.138	0.523	18.844	0.211
TV regurgitation	52 (73%)	4 (80%)	1.704	0.189	15.371	0.635

**Prediction of Distal Aortic Repair**	**Multivariate Cox Regression Analysis**	
	**Hazard Ratio**	***p***
Diameter of ascending aorta (cm)	0.800	0.968
Diameter of descending aorta (cm)	7.853	0.850
Diameter of abdominal aorta (cm)	18.369	0.436

ACEi, angiotensin-converting enzyme inhibitors; ARB, angiotensin receptor blockers; AV, aortic valve; BAB, beta-adrenergic blockers; BMI, body mass index; BSA, body surface area; CI, confidence interval; HDL, high-density lipoprotein; LDL, low-density lipoprotein; MV, mitral valve; *n*, number of events; TV, tricuspid valve. If less than total, we present the number of individuals with available information behind a slash. [1] Two women in the MFS cohort with aortic dissection during pregnancy were excluded from long-term analysis.

Table S8. Long-term aortic growth in individuals with Marfan syndrome (MFS) and with bicuspid aortic valve disease (BAV).

		**Multiple regression analysis**		
**Aortic Segment**	**Variable**	**Estimate (mm)**	**Standard Error**	***p***
**Aortic root**	Baseline diameter (mm)	-1.77	0.90	0.300
**(*N* = 51)**	Follow-up time (years)	0.64	0.19	0.002
	Presence of MFS	1.91	1.33	0.156
	Previous pregnancy	-0.20	0.93	0.835
**Ascending aorta**	Baseline diameter (mm)	-2.13	1.01	0.040
**(*N* = 50)**	Follow-up time (years)	0.16	0.18	0.367
	Presence of MFS	-3.72	1.41	0.012
	Previous pregnancy	0.57	0.86	0.514

**Descending aorta**	Baseline diameter (mm)	0.56	0.83	0.504
**(*N* = 64)**	Follow-up time (years)	0.01	0.15	0.953
	Presence of MFS	-0.06	1.30	0.966
	Previous pregnancy	0.01	0.75	0.991
**Abdominal aorta**	Baseline diameter (mm)	1.74	0.86	0.048
**(*N* = 47)**	Follow-up time (years)	-0.06	0.30	0.832
	Presence of MFS	0.74	3.02	0.806
	Previous pregnancy	-0.34	1.26	0.789

N, number of individuals with available diameters.
